# Quality of Life (QoL) and Associated Factors Among Long Term Recipients of Buprenorphine-Naloxone (Bpn-N) Maintenance Treatment

**DOI:** 10.1192/j.eurpsy.2026.10807

**Published:** 2026-07-31

**Authors:** P. Thapa

**Affiliations:** Psychiatry, Nobel Medical College Teaching Hospital, Biratnagar, Nepal

## Abstract

**Introduction:**

Opioid dependence adversely affects various aspects of life. Agonist maintenance treatment has been found to be most effective for opioid dependence in terms of substance use parameters and quality of life. The current study focusses at the treatment satisfaction and quality of life of those on long term Buprenorphine-naloxone (BPN-N) treatment at a national level addiction treatment centre in India.

**Objectives:**

TO MEASURE QUALITY OF LIFE (QOL) AND ASSOCIATED FACTORS AMONG LONG TERM RECIPIENTS OF BUPRENORPHI(BPN-N) MAINTENANCE TREATMENT

**Methods:**

This cross-sectional, observational study included 130 opioid-dependent males on BPN-N maintenance treatment for ≥2 years at the Outpatient setting of the National Drug Dependence Treatment Centre (NDDTC), AIIMS, New Delhi, India using purposive sampling. Participants were assessed using Scale to assess satisfaction with medications for addiction treatment- Buprenorphine Naloxone for Heroin Addiction (SASMAT-BUNHER SCALES) and Opioid Substitution Treatment QOL questionnaire (OST-QOL). Institute ethics clearance was obtained.

**Results:**

Participants were on treatment for > 5years, mean age (40 years), married (73.1%), employed (96%), literate (77%), upper lower socio-economic status (64%) and > 85% reported treatment satisfaction as high to excellent on various domains. More than 70% of them achieved higher scores (≥3) in various domains of QOL including Personal Development and Discrimination. Significant positive association was observed between older age, higher socio-economic status, higher education, married, patient satisfaction and high QOL (p value <0.005). No association was found with employment status, BPN dose and treatment duration.

**Conclusions:**

Long term BPN-N maintenance treatment recipients report high level of satisfaction with treatment and good quality of life in various domains.

**Disclosure of Interest:**

None Declared

